# Inhibitory binding to plastoquinone B site on D1 protein of photosystem II leading to decreased population growth rate via disruption of cellular energy metabolism

**DOI:** 10.3389/fpls.2026.1910560

**Published:** 2026-07-31

**Authors:** Li Xie, Knut Erik Tollefsen

**Affiliations:** Norwegian Institute for Water Research (NIVA), Ecotoxicology and Risk Assessment, Oslo, Norway

**Keywords:** adverse outcome pathway, growth inhibition, photosystem II inhibition, SeqAPASS, weight of evidence

## Abstract

Photosystem II (PSII) is essential for photosynthesis in primary producers, facilitating the primary photochemical reaction to oxidize water and drive the production of ATP and NADPH. A critical interaction within PSII occurs at the plastoquinone B (QB) site on the D1 protein, where electron transfer from QA to QB ensures the continuity of photosynthetic electron flow and energy transduction. PSII-inhibitors, such as diuron, can directly bind to the QB site of the D1 protein, thus hindering the flow of electrons and reducing the synthesis of ATP and other compounds (e.g. NADP) in chloroplasts. Such disruption would constrain downstream processes on multiple biological levels, ultimately leading to growth inhibition. PSII inhibitors are typically used as herbicides that are commonly detected in surface and ground waters, especially in agricultural regions. Their mobility in surface water is associated with acute and chronic effects in non-target organisms and especially primary producers such as plants, algae, and aquatic macrophytes that have conserved QB binding sites similar to target weeds. To improve mechanistic understanding and strengthen ecological risk assessment of PSII-inhibitor (e.g. herbicides), AOP #567 was developed. This AOP outlines a linear cascade of events, beginning with the binding of specific PSII inhibitors to the QB site on the D1 protein, leading to successive reductions in PSII efficiency, photosynthesis, mitochondrial OXPHOS, and ATP production, resulting in reduced growth at the individual level, and culminating in decreased population growth rate in primary producers. By systematically linking molecular interactions to population-level outcomes, AOP #567 provides a transparent and biologically plausible framework to assess hazard and aid environmental risks of PSII-inhibitors.

## 1 Introduction and background

Photosynthesis is the fundamental biological process to convert solar energy into chemical energy and store it as carbohydrates in plants, algae, and cyanobacteria. This process supports primary productivity in both terrestrial and aquatic ecosystems. Being at the centre of photosynthesis, photosystem II (PSII) performs the most important photochemical reaction through light capture resulting in oxidation of water molecules that release oxygen, protons and electrons (Chen et al., 2007). Such electrons are then passed on to the photosynthetic electron transport pathway, and eventually lead to the generation of ATP and NADPH. Within the PSII complex, plastoquinone B (Q_B_) binds at the Q_B_ site of the D1 protein, where it accepts electrons from plastoquinone A (Q_A_) (Ohad and Hirschberg, 1992). This step is an essential electron transfer between Q_A_ and Q_B,_ which is essential for electron flow and energy transduction in photosynthesis (Vermaas et al., 1984). The binding site of the Q_B_ is a common molecular target for many herbicides because it’s central role in photosynthetic electron transport (Fuerst and Michael, 1991).

PSII-inhibiting herbicides exert their phytotoxic effects by competitively binding to the QB site of the D1 protein, thereby blocking electron transfer between QA and QB. This interaction disrupts linear electron flow from PSII to photosystem I (PSI), suppressing the formation of ATP and NADPH in the chloroplast. As a result, the first stage of carbon fixation and carbohydrate production has been impaired (Wilkinson et al., 2015). A reduction in carbon fixation will decrease the supply of respiratory substrates and modify the redox conditions as well as the energy transfer in chloroplasts and mitochondria, thereby affecting mitochondrial oxidative phosphorylation (OXPHOS) and ATP production (Amthor and Baldocchi, 2001; Hanson et al., 2023; Igamberdiev and Bykova, 2023). However, this relationship are not always linear, because mitochondrial respiration may be maintained, reallocated, or transiently enhanced as part of cellular acclimation under some stress conditions (Cardol et al., 2003; Hoefnagel et al., 1998; Xue et al., 1996). A decrease in the supply of ATP may reduce the energy available for biosynthesis, cell division and other necessary activities of the cell in the growth stage (Forbes and Calow, 1999). In primary producers, reduced individual growth is observed as lower biomass accumulation, reduced cell division rates, and decreased shoot or frond development, depending on the organism (Negri et al., 2015; Nestler et al., 2012). Consistent reductions in individual growth across a population can lead to a decline in overall population growth rate (Forbes and Calow, 2002).

PSII inhibitors are commonly used as herbicides in agriculture and represents an environmental concern due to high toxic potency, high mobility, stability in terrestrial and aquatic ecosystems (Brock et al., 2000). These chemicals are often found in surface and ground water as they are introduced through agricultural run-off or leaching from treated soils. In addition, some PSII inhibitors have non-agricultural uses, including direct use in aquaculture to manage cyanobacterial blooms and off-flavour episodes, and use as industrial or marine biocides in antifouling coatings and paints/material preservatives, thus providing additional emission pathways to the aquatic environments (U.S. EPA, 2015; Gatidou et al., 2007; Konstantinou and Albanis, 2004; Schrader, 2022; Thomas, 2001). Primary producers such as algae, aquatic macrophytes, and terrestrial plants are particularly susceptible to PSII inhibitors because the QB binding site on the D1 protein is highly conserved across oxygenic photosynthetic organisms, including both non-target primary producers and the weed species these herbicides are designed to control (Oettmeier, 1999; Battaglino et al., 2021). Even at low environmental concentrations as 0.001–10 µg/L, Diuron can still negatively affect photosynthetic efficiency, growth rates and normal primary productivity (Wilkinson et al., 2015; Battaglino et al., 2021; Oettmeier, 1999). These effects may cascade through ecosystems and change species composition, community structures, and stability and functioning of whole ecosystems. Despite well-developed knowledge above the effects of PSII-inhibitors, structured and causative description of the linkage between molecular changes and adverse effects at the organism and population level are generally lacking. To improve mechanistic insights and risk assessment of herbicides that inhibit photosystem II, the Adverse Outcome Pathway (AOP) #567 (Binding to plastoquinone B site leading to decreased population growth rate via photosystem II inhibition) was developed to illustrate how PSII inhibitors disrupting the D1 protein function may interfere with photosynthetic electron transport and reduce population growth in primary producers. AOP#567 organizes dispersed evidence on PSII-inhibitor effects into a transparent, evidence-rated pathway that links a well-defined molecular target to bioenergetic impairment and growth-related adverse outcomes in primary producers.

## 2 AOP description

**Figure 1:**
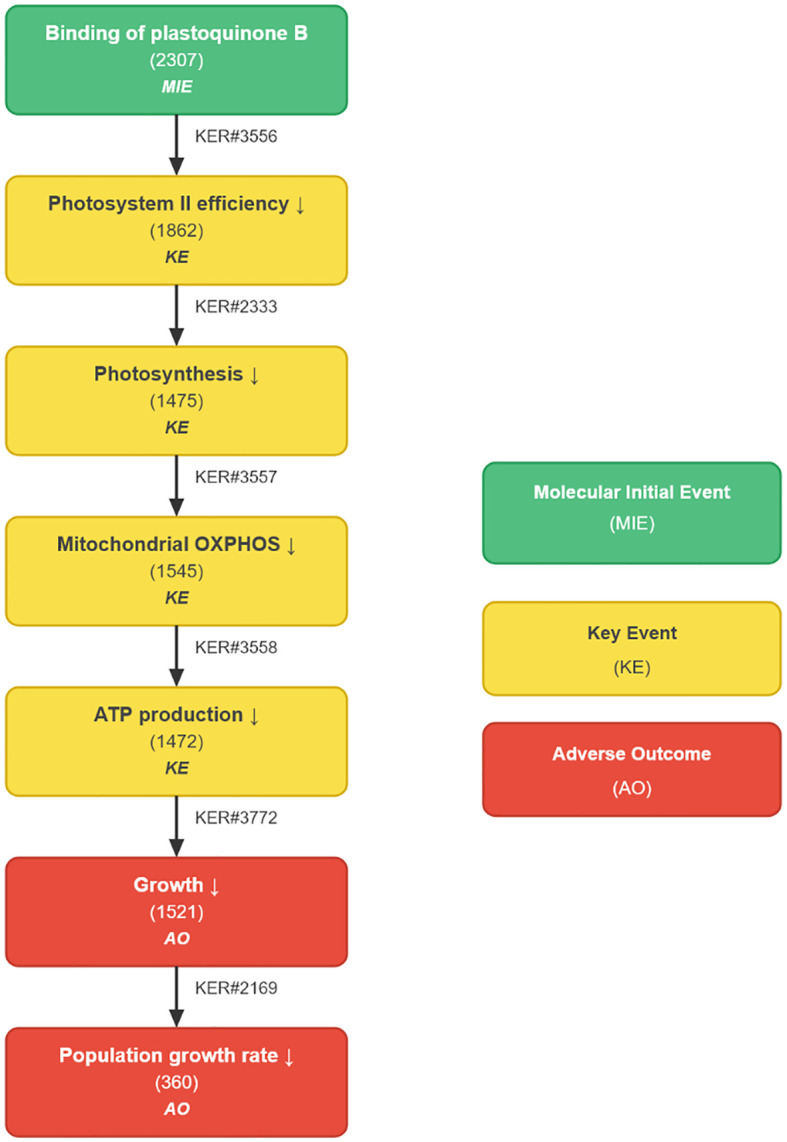
Graphical representation of AOP#567 linking inhibitory binding of PSII inhibitors to the plastoquinone B (QB) site on the D1 protein of photosystem II to decreased population growth rate in primary producers. The pathway includes the molecular initiating event (MIE#2307), binding of PSII inhibitors to the QB site of the D1 protein; key event (KE#1862), decreased photosystem II efficiency; key event (KE#1475), decreased photosynthesis; key event (KE#1545), decreased mitochondrial oxidative phosphorylation; key event (KE#1472), decreased ATP production; adverse outcome (AO#1521), decreased individual growth; and adverse outcome (AO#360), decreased population growth rate. Numbers in parentheses indicate AOP event IDs and key event relationship (KER) IDs.

The present AOP (**Figure 1**) describes a biologically plausible sequence of events initiated by the binding of PSII inhibitors to the QB site of the D1 protein within the PSII complex (MIE#2307). This molecular initiating event is linked to reduced PSII efficiency (KE#1862), decreased photosynthesis (KE#1475), impaired mitochondrial oxidative phosphorylation (KE#1545), and reduced ATP production (KE#1472). These changes can lead to decreased growth at the individual level (AO#1521) and ultimately to reduced population growth rate (AO#360) in primary producers (**Table 1**). These two adverse outcomes are retained as separate events because individual growth describes organism-level performance, whereas population growth rate describes change at the population level. The extent to which they can be measured independently depends on organism type and toxicity test being used.

**Table 1:** Summary of key events in AOP #567 and related measurement methods.

Event ID	Description	Measurement methods	Reference
2307	Binding of PSII inhibitors to plastoquinone B	QSAR, molecular docking studies, radioligand binding, fluorescence-based assays, surface plasmon resonance, isothermal titration calorimetry, resistance mutant test.	(Arnaud et al., 1994; Battaglino et al., 2021; Broser et al., 2011; Giardi and Pace, 2006; Piletska et al., 2006; Sundby et al., 1993; Tischer and Strotmann, 1977; Zimmermann et al., 2006)
1862	Decrease, photosystem II efficiency	Chlorophyll fluorescence analysis (F_v_/F_m_ ratio), oxygen evolution rates, PAM fluorometry, modulated fluorometry, D1 protein degradation assays	(Alfonso et al., 1996; Delieu and Walker, 1981; Maxwell and Johnson, 2000; Xia et al., 2023)
1475	Decrease, photosynthesis	Carbon fixation rates (^14^C uptake), oxygen production, infrared gas analysis (CO2 uptake), measurement of Rubisco activity, O_2_ evolution	(Grant and Howard, 1980; Lilley and Walker, 1974; Milligan et al., 2015; Sales et al., 2020; van Gorkom and Gast, 1996; Xie et al., 2019)
1545	Decrease, mitochondrial OXPHOS	Respirometry, ATP synthesis rates, enzyme activity assays for complexes I-IV, mitochondrial membrane potential assays, blue-native PAGE	(Coulson et al., 2024; Djafarzadeh and Jakob, 2017; Hitchins et al., 2001; Lundin et al., 1976; Xie et al., 2019; Yan and Forster, 2009)
1472	Decrease, ATP production	Luciferase-based ATP assays, enzymatic assays for ATP synthase, NMR spectroscopy, high-performance liquid chromatography (HPLC)	(Allakhverdiev et al., 2005; Coulson et al., 2024; Hitchins et al., 2001; Juarez-Facio et al., 2021)
1521	Decrease, Growth	Biomass measurements, frond number counts, cell size analysis, shoot/root length, specific growth rate in culture, optical density	Negri et al. (2015); Nestler et al. (2012); Xie et al. (2019)
360	Decrease, population growth rate	Growth rate calculations from biomass or cell count, specific growth rate in cultures, flow cytometry for cell cycle analysis, optical density measurements	(Peniuk et al., 2016; Eppley, 1972; Xie et al., 2025, 2019). OECD guidelines

The MIE constitutes the competitive binding of PSII inhibitors to the QB site of the D1 protein, which disrupts the transfer of electrons between QA and QB. This blockage has a direct inhibitory impact on the functioning of the PSII complex and causes the first downstream KE: the inhibition of PSII efficiency, which is typically reported as Fv/Fm ratio or PhiPSII. Lower PSII efficiency indicates reduced energy conversion in the light-dependent reactions of photosynthesis. This can decrease carbon fixation and carbohydrate synthesis, reducing the supply of respiratory substrates to mitochondria and altering the redox and energy exchange between chloroplasts and mitochondria. KER#3557 is therefore retained as an intermediate bioenergetic relationship, but it is treated as context-dependent because mitochondrial OXPHOS may be constrained, maintained, or reallocated depending on acclimation state and stress conditions. Reduced ATP availability can then limit the energy supply for biosynthesis, cell division, and maintenance processes that support individual growth. When individual growth is consistently reduced across organisms in a population, the overall population growth rate declines as a consequence.

This series of KEs is the core pathway used here to describe how a molecular-level interaction with a chemical stressor can produce an ecologically relevant adverse outcome. Each KE and adjacent KER is evaluated using biological plausibility, empirical evidence, dose-response relationships, and taxonomic concordance, as elaborated in the section “Summary of scientific evidence assessment” below. The pathway is not intended to imply that every intermediate event changes in a strictly proportional or irreversible manner under all conditions. In particular, KER#3557 is retained because chloroplast-mitochondrial metabolic coupling is well supported, but it is assigned moderate confidence because compensatory respiratory responses may modify the magnitude or direction of this relationship. Secondary effects, such as oxidative stress and thylakoid membrane damage, can also occur after PSII disruption, but they are considered modifying processes rather than required components of this AOP.

## 3 AOP development strategy

The development of AOP#567 followed the guidance outlined in the OECD Users’ Handbook Supplement to the Guidance Document for Developing and Assessing AOPs (OECD, 2018). The pathway was constructed using a structured, evidence-based, and transparent process integrating systematic literature review, expert consultation, and formal weight-of-evidence (WoE) evaluation consistent with modified Bradford Hill considerations.

### 3.1 Problem formulation and AOP scoping

This AOP describes a well-characterized toxicity mechanism that has been widely exploited to control herbaceous pests. However, due to the high degree of conservation of the molecular MIE, KEs, and AOs across other primary producers (e.g., microalgae, macroalgae, and vascular plants), the potential for adverse effects in non-target species is of concern. This specific AOP was initiated based on the well-characterised mode of action (MOA) of PSII-inhibiting chemicals that interact with the Q_B_ binding niche of the D1 protein. The MIE was defined as interference at the Q_B_ site within PSII. Downstream key events (KEs) and the adverse outcome (AO) were identified sequentially based on established physiological and biochemical links between photosynthetic performance, cellular energy metabolism, individual growth, and population-level effects. The proposed sequence comprises decreased photosystem II (PSII) efficiency, reduced photosynthesis, impaired mitochondrial oxidative phosphorylation (OXPHOS), decreased ATP production, reduced growth, and decreased population growth rate. The corresponding key event relationships (KERs) are KER#3556, KER#2333, KER#3557, KER#3558, KER#3772, and KER#2169, respectively.

### 3.2 Systematic literature identification and screening

A tiered literature identification and screening process was employed for the collection and filtering of literature to establish an evidence base for AOP #567. Literature identification was performed using AOP-helpFinder (v3.0), a PubMed-based text-mining tool that identifies stressor-event and event-event co-occurrences relevant to AOP development (Jornod et al., 2022; Jaylet et al., 2023, 2025). A curated list of key-event terms and alternative terms was submitted to AOP-helpFinder to capture literature related to the molecular initiating event, key events, adverse outcome, and adjacent key event relationships. The search strategy combined stressor terms, event-related terms and taxa terms (**Supplementary Information**; Search Terms). After retrieval, redundant hits across overlapping event terms were removed following the overlap-analysis strategy of Xie and Tollefsen (2025). Swift-Reviewer (Version 1.43.1063) was then used to refine the evidence corpus through structured tagging, relevance scoring, and confidence ranking. The remaining records were mapped to the corresponding KEs, and screened for relevance before full-text assessment. Only studies that employ photosynthetic organisms or mechanistically related primary-producer systems and furnish experimental, quantitative or mechanistic data on the defined MIE, KEs, AO, or adjacent KERs were selected. Studies that did not have a defined endpoint, involve a bioenergetically significant photosynthetic system, provide traceable primary evidence, or contain sufficient full-text and endpoint information were excluded.

### 3.3 Weight-of-evidence evaluation for key events relationships

Modified Bradford-Hill considerations were usded for AOP WoE evaluation, following AOP Developer Handbook.developer handbook (v2.8) (Becker et al., 2015; Collier et al., 2016; Villeneuve et al., 2026). For each KER, evidence was evaluated by: (1) biological plausibility on the basis of on known photosynthetic and bioenergetic principles. (2) the essentiality of individual KEs was determined by examining whether prevention or attenuation of an upstream KE results in reduction or absence of the downstream KE. (3) empirical evidence was evaluated based on dose-response consistency, temporal concordance, and incidence concordance across available studies. For each criterion, the level of support was classified as high, moderate, or low using explicit criteria. High support required a direct mechanistic basis together with pathway-relevant experimental evidence showing dose-response, temporal, or incidence concordance in a relevant primary-producer system. Moderate support was assigned when the relationship was mechanistically plausible and supported by indirect experimental evidence, related biological systems, or incomplete quantitative concordance. Low support was assigned when evidence was mainly conceptual, associative, based on different stressors or systems, or lacked paired measurements of the upstream and downstream events. AOP-helpFinder co-occurrence scores were used as supplementary support for assessing biological plausibility by identifying studies reporting co-occurrence of the defined KEs, but they were not considered direct evidence of mechanistic or causal relationships. Final WoE ratings were based on manual evaluation of study relevance, endpoint specificity, pathway concordance, and uncertainty (**Supplementary Information**; Bioplau/Essentiality/KE method/Empirical/Concordance table).

### 3.4 SeqAPASS

SeqAPASS was used to evaluate taxonomic conservation of the D1 protein, the molecular target for PSII inhibitors in AOP #567 (LaLone et al., 2016). The *Chlamydomonas reinhardtii* photosystem II protein D1 sequence (NP_958377.1) was used as the query in SeqAPASS v9.1 (**Supplementary Information**; SeqAPASS_Level1/2/3_Primary_Report) Level 1 analysis used default settings, including a 56.27% similarity cut-off based on the first local minimum in the percent-similarity distribution and the next ortholog candidate above that minimum, and an E-value threshold of 0.01. Results were sorted by taxonomic class, species read-across was enabled, and searches were not restricted to eukaryotes or marine species. Level 2 functional domain analysis was conducted using the CHL0003 domain, annotated as psbA/photosystem II protein D1. Taxonomic groups were evaluated using species representation, mean and median percent similarity, and SeqAPASS susceptibility prediction. Level 3 residue analysis used NP_958377.1 as the template sequence and SeqAPASS-prioritized accessions from Level 1. After COBALT alignment, D1 residues Y254, F255, S264, and L271, representing the QB/herbicide-binding region, were selected for evaluation. Level 3 results were interpreted using the “Similar Susceptibility as Template” prediction, Yes(Y)/No(N) species counts, and residues observed at the selected positions. Groups with majority “Y” predictions and conservation of the Y254, F255, S264, and L271 pattern were considered to have residue-level support for intrinsic molecular susceptibility. Groups with gaps, unknown residues, or alternative amino acids were interpreted with greater uncertainty.

## 4 Summary of scientific evidence assessment

### 4.1 Biological plausibility of the key event relationships

**Figure 2:**
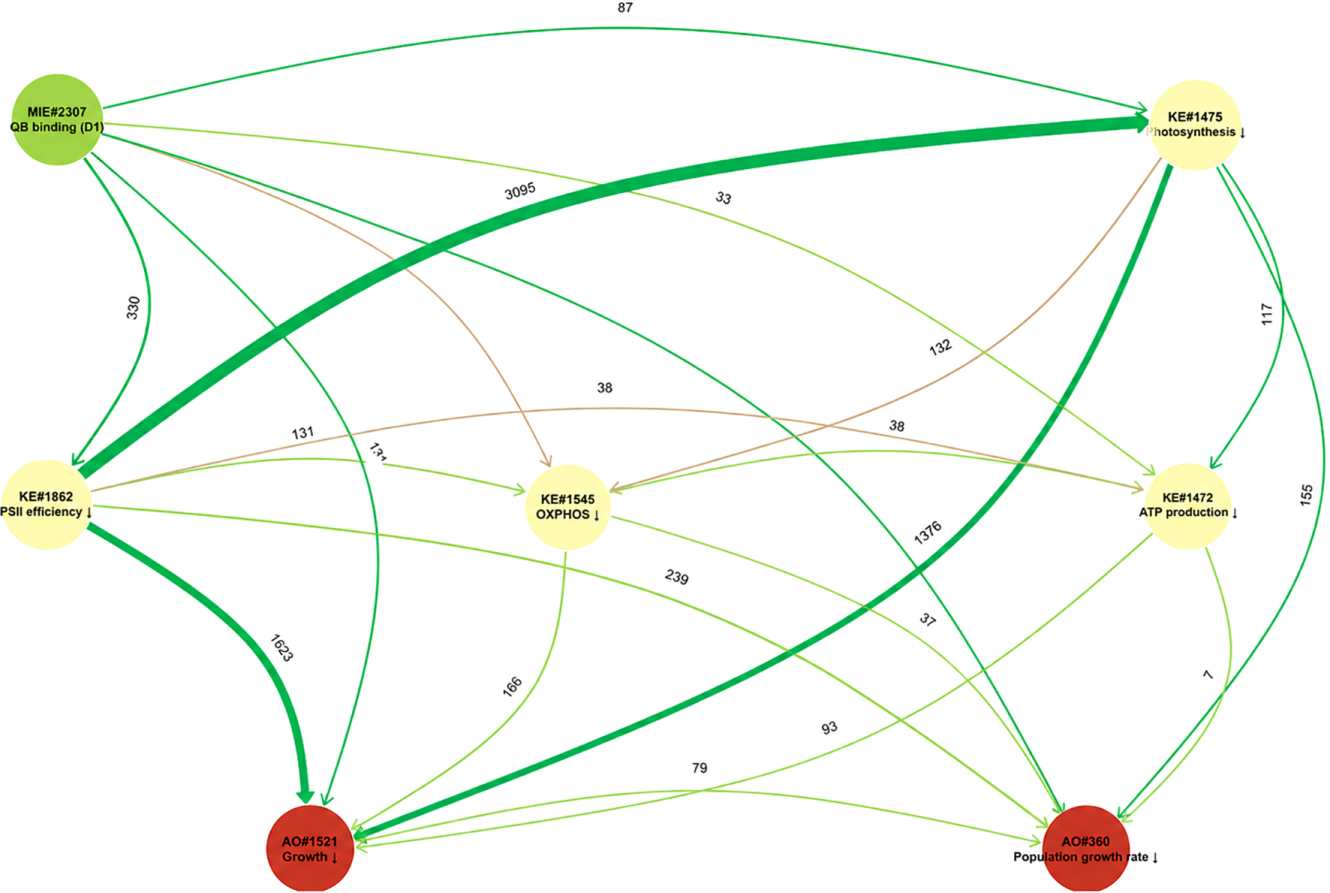
Literature-support network for AOP#567 key event relationships (KERs) generated from AOP-helpFinder screening. Arrow thickness represents the Count Score (CS), defined as the number of publications in which the paired upstream and downstream events co-occurred. Arrow colour represents the AOP-helpFinder confidence category: dark green = very high, light green = moderate, and brown = low. Node colour denotes event type: green = molecular initiating event (MIE), yellow = key event (KE), and red = adverse outcome (AO). The literature co-occurrence scores were used to prioritize evidence review and should not be interpreted alone as proof of causality.

Biological plausibility means the known structural, or functional correlations between upstream key event and downstream key event in unperturbed biology or normal conditions (OECD, 2018). Such basic knowledge represents a basis for extrapolation or hypothesis-generation of the probable effects of a biological perturbation induced by a stressor. In addition to mechanistic reasoning and primary literature, AOP-helpFinder (v3.0) was used to map publication co-occurrence for each KER in AOP#567, but these co-occurrence results were interpreted only as literature-support and source for prioritization rather than as proof of causality (**Figure 2**).

The biological plausibility of KER#3556 (Binding of plastoquinone B leads to Decrease in Photosystem II efficiency) is considered high. Under normal photosynthetic function, movement of electrons from Q_A_ to Q_B_ in the D1 protein is a necessary step in the photochemistry of PS II. Therefore, the interference at the QB site will block electron transfer past the Q_A_ and result in a reduction in the efficiency by which PSII converts the energy of absorbed light into photochemical energy. This causal linkage is direct, structurally well defined, and strongly supported by the canonical understanding of PSII function and its herbicide-binding niche (Battaglino et al., 2021; Ohad and Hirschberg, 1992; Tietjen et al., 1991). This relationship is further supported by AOP-helpFinder, which identified 330 publications reporting co-occurrence of Q_B_ binding and decreased PSII efficiency, with a very high relevance score, reflecting a strong and well-documented literature basis for this relationship.

The biological plausibility of KER#2333 (Decrease in Photosystem II efficiency leads to Decrease in Photosynthesis) is also high. PSII efficiency is a measure of the efficiency of the light reactions in terms of linear electron transport. When the PSII efficiency is decreased, it is inevitable that generation of reducing equivalents and proton motive force needed to support downstream photosynthetic activities will be reduced. Mechanistically, it would then be expected that impaired PSII function would directly translate into overall impaired photosynthetic performance. This association is the core principle of the photosynthesis process which leads to the use of fluorescence-based PS2 measurements as operationally useful characteristics of photosynthetic capability (Baker, 2008; Maxwell and Johnson, 2000). AOP-helpFinder identified 3095 publications co-reporting decreased PSII efficiency and decreased photosynthesis with a very high relevance score, confirming that this is the best-supported relationship in AOP#567.

The biological plausibility of KER#3557 (Decrease in Photosynthesis leads to Decrease in mitochondrial oxidative phosphorylation) is considered moderate. In photosynthetic eukaryotes, chloroplasts and mitochondria are metabolically coupled through exchanges of carbon intermediates, reductant, ATP demand, and redox signals (Igamberdiev and Bykova, 2023). Reduced photosynthetic carbon assimilation can decrease respiratory substrate supply and alter cellular redox balance, thereby constraining mitochondrial oxidative metabolism. However, this relationship is context dependent. Mitochondrial respiration may be maintained, reallocated, or transiently enhanced under some stress conditions as part of compensatory acclimation, rather than declining in direct proportion to photosynthesis (Cardol et al., 2003; Hoefnagel et al., 1998; Xue et al., 1996). KER#3557 is therefore retained as an important but non-linear intermediate relationship, supported by chloroplast-mitochondrial interaction studies and treated as moderate rather than high confidence (Cardol et al., 2003; Hoefnagel et al., 1998; Padmasree and Raghavendra, 2001; Rebeille and Gans, 1988; Vera-Vives et al., 2024). AOP-helpFinder identified 76 publications for this KER with a moderate relevance score, consistent with a plausible, but less directly documented relationship than the upstream photochemical KERs.

The biological plausibility of KER#3558 (Decrease in mitochondrial oxidative phosphorylation leads to Decrease in ATP production) is high. Oxidative phosphorylation is the canonical process by which mitochondrial electron transport generates a proton gradient that drives ATP synthesis. A depletion in mitochondrial oxidative phosphorylation will consequently mean a depletion in the capacity to generate ATP unless substituted by other energy-generating mechanisms. This relationship is not only one of the fundamental principles of cellular bioenergetics, but also regarded as a principle that has been preserved in eukaryotic systems (Brand and Nicholls, 2011; Mitchell, 1961; Wilson, 2017). However, the AOP-helpFinder identified 76 publications with a moderate relevance score, indicating the limited number of studies simultaneously measuring both mitochondrial OXPHOS and ATP production in primary producers. Nevertheless, the mechanistic link between OXPHOS and ATP synthesis is a fundamental and well-established principle of cellular bioenergetics (Boyer, 1997; Mitchell, 1961).

The biological plausibility of KER#3772 (Decrease in ATP production leads to Decrease in individual growth) is considered high. ATP is the primary energy currency of the cell and is required for biosynthesis, cell division, ion transport, and maintenance of cellular processes that collectively support organismal growth. In primary producers, individual growth depends on continuous ATP supply to drive the synthesis of proteins, nucleic acids, lipids, and cell wall components. When ATP production is reduced, the energy available for these anabolic processes declines. This constrains cell division rates, biomass accumulation, and the development of new tissue, all of which are measurable components of individual growth. This relationship is well supported by fundamental principles of cellular bioenergetics and is consistent across a wide range of photosynthetic organisms including microalgae, macrophytes, and higher plants (Hardie, 2007; Nestler et al., 2012; Vera-Vives et al., 2024). However, AOP-helpFinder identified 93 publications for this KER with a moderate relevance score. The moderate score likely reflects the fact that studies reporting both ATP depletion and individual growth endpoints simultaneously in primary producers are less common than those reporting each endpoint.

The biological plausibility of KER#2169 (Decrease in individual growth leads to Decrease in population growth rate) is high because the link between reduced individual growth and reduced population growth rate is a fundamental demographic relationship. In multicellular primary producers, impaired individual growth can be expressed as reduced biomass accumulation, frond area, shoot length, or tissue production, which can subsequently affect reproduction, recruitment, survival, and competitive capacity. These changes can lower population-level parameters such as the intrinsic rate of increase (r), net reproductive rate (R0), gamete production, frond number, or total population biomass. In unicellular algae, the distinction between individual and population growth is less clear because cell division contributes directly to both individual performance and (sub)population increase. For this KER, the AOP-helpFinder identified 79 publications with a moderate relevance score. However, the moderate score likely reflects the broad ecological and demographic literature from which this relationship draws, rather than a weak mechanistic connection, as this relationship is one of the most fundamental in population ecology and is well supported across taxa (Forbes and Calow, 1999, 2002; Xie et al., 2019).

Overall, the strongest biological plausibility in AOP#567 lies in the early KERs (KER#3556 and KER#2333), because these relationships follow directly from highly conserved and well-characterized photosynthetic mechanisms. The downstream KERs (KER#3557, KER#3558, KER#3772, and KER#2169) remain biologically credible and supported by bioenergetic theory, but they are more context dependent. KER#3557 is an exemplary of this patten, which operates through organelle cross-talk and whole-cell metabolic integration rather than a single direct photochemical step.

### 4.2 Essentiality of the key events

Evidence for essentiality in an AOP refers to demonstrating that an upstream event is required for the activity of an downstream event to occur (Villeneuve et al., 2014). This is typically established through experimental manipulation showing that when an upstream event is prevented, attenuated, or reversed, the downstream event is also prevented, reduced, or returns toward normal. Together, these approaches help to determine whether a KE is causally necessary for progression through the pathway. The essentiality of the KEs in AOP#567 is overall supported at a moderate to high level, with the strongest evidence available for the early photochemical KEs and comparatively weaker, though still supportive, evidence for the later bioenergetic KEs.

In this assessment, evidence was distinguished between pathway-specific essentiality evidence, where a key event was manipulated or reversed within the PSII-inhibitor pathway, and broader biological support, where evidence came from related stressors, genetic perturbations, or general bioenergetic studies. The latter was used to support biological relevance, but was not treated as direct proof that the full proposed pathway is trigger by PSII-inhibitor exposure.

The essentiality of the MIE# 2307 (Binding of plastoquinone B within D1 protein of PSII) is considered high, because competitive binding studies demonstrate that PSII herbicides act specifically through interaction with the QB site of the D1 protein, and that this interaction is required to initiate inhibition of PSII electron transport (Battaglino et al., 2021; Sundby et al., 1993).

The essentiality of KE#1862 (Decrease in PSII efficiency) is considered high, as supported by direct exposure-recovery evidence showing that short-term diuron exposure reduced PSII efficiency in the seagrass *Zostera capricorni*, and removal of exposure allowed recovery of photochemical performance, demonstrating that this KE is required for downstream photosynthetic impairment (Macinnis-Ng and Ralph, 2003).

The essentiality of KE#1475 (Decrease in Photosynthesis) is considered high. Chronic exposure to diuron caused sustained photosynthetic impairment in tropical seagrasses, and transfer to clean water enabled recovery of both photosynthesis and growth-related endpoints, supporting reduced photosynthesis as a necessary upstream determinant of later adverse responses (Negri et al., 2015).

The essentiality of KE#1545 (decreased mitochondrial OXPHOS) is considered low because direct evidence from studies specifically designed to test PSII inhibitors across the complete proposed pathway is limited. Indirect support is available from photosynthetic organisms, including Chlamydomonas reinhardtii and Physcomitrium patens, showing that disruption of mitochondrial respiration can reduce cellular ATP levels and alter energy metabolism (Cardol et al., 2003; Rebeille and Gans, 1988; Vera-Vives et al., 2024). These studies support mitochondrial OXPHOS as a biologically relevant intermediate key event, but they should be interpreted as broader bioenergetic support rather than pathway-specific essentiality evidence for PSII-inhibitor exposure.

The essentiality of KE#1472 (Decrease in ATP production) is considered moderate. Genetic knockout and inhibition-rescue evidence indicate that ATP production is required for growth-related processes: knockout of the mitochondrial ATP synthase subunit FAd in Physcomitrium patens caused drastic growth reduction with only minor photosynthetic changes, and supplying ATP as the sole phosphorus source in Prorocentrum donghaiense restored growth from 0.09 to 0.36 d-1 (Vera-Vives et al., 2024; Lapaille et al., 2010; Li et al., 2015). However, these studies do not directly test ATP depletion downstream of PSII inhibition across the complete AOP sequence. They are therefore used as general biological support for the growth relevance of ATP limitation, rather than as direct pathway-specific evidence.

The essentiality of AO#1521 (Decrease in individual growth) is considered high, as supported by direct exposure-recovery evidence showing that diuron-exposed *Lemna minor* and *Lemna gibba* recovered individual growth after transfer to clean media, confirming that attenuation of upstream energy-related KEs reversed individual growth suppression. In clonal macrophytes and unicellular algae, individual growth is the direct mechanism for population reproduction, making individual growth inhibition structurally necessary for population growth rate suppression (Burns et al., 2015; Forbes and Calow, 2002).

### 4.3 Empirical support for the key event relationships

Empirical support for the KERs in AOP#567 is overall moderate to high, with the strongest evidence for the upstream photochemical relationships. Following OECD guidance, support was evaluated based on dose-response consistency (the upstream KE is impacted at a concentration or dose equal to or lower than that which impacts the downstream KE), temporal concordance (the upstream KE occurs before or at same time the downstream KE is observed) and incidence concordance (the upstream KE is observed in an equal or greater proportion of the test population compared to the downstream KE). These empirical ratings were assigned by manual WOE evaluation of the available studies.

KER 3556 (Binding of plastoquinone B within D1 protein of PSII lead to Decrease in PSII efficiency) has high empirical support. Tischer and Strotmann (1977) showed that binding constants and inhibition constants were closely matched in broken chloroplasts, supporting direct mechanistic concordance between target-site occupancy and PSII inhibition. This relationship is reinforced by Battaglino et al. (2021), who demonstrated concentration-dependent inhibition of PSII function in isolated pea thylakoids, with diuron acting most potently (IC_50_ = 0.0718 - 0.0802 µM), followed by terbuthylazine and metribuzin (IC_50_ = 0.110–0.156 µM), whereas metobromuron and bentazon required much higher concentrations. These studies provide strong dose-response evidence for a direct linkage between the MIE and reduced PSII efficiency.

KER 2333 (Decrease in PSII efficiency leads to Decrease in photosynthesis) also has high empirical support. Genty et al. (1989) demonstrated that ΔF/Fm′ is directly proportional to the quantum yield of non-cyclic electron transport and closely related to CO_2_ assimilation across a wide range of light and CO_2_ conditions, providing strong response-response support. Under chemical stress, Flores et al. (2021) showed that diuron reduced ΔF/Fm′ in *Acropora millepora* at lower concentrations than net photosynthesis, with EC_50_ values for ΔF/Fm′ near 2 µg/L compared with a net photosynthesis EC_50_ of 19.4 µg/L, indicating that reduced PSII efficiency precedes measurable declines in net photosynthetic performance.

KER 3557 (Decrease in photosynthesis leads to Decrease in mitochondrial oxidative phosphorylation) has moderate empirical support. Xue et al. (1996) demonstrated that inhibition of photosynthesis reduced light-enhanced dark respiration (LEDR), a respiratory response that depends on prior photosynthetic activity in *Chlamydomonas reinhardtii*. Exposure to diuron and glycolaldehyde consistently decreased LEDR across the tested illumination conditions, indicating a relationship between photosynthetic activity and subsequent respiratory stimulation. However, this evidence does not establish a universally linear decrease in mitochondrial OXPHOS after photosynthesis is reduced. Instead, it supports chloroplast-mitochondrial coupling while leaving room for compensatory respiratory responses under some stress conditions. The evidence base for this KER remains less extensive than for the preceding relationships, and quantitative paired datasets directly linking reduced photosynthesis to decreased oxidative phosphorylation remain limited.

KER 3558 (Decrease in mitochondrial oxidative phosphorylation leads to Decrease in ATP production) has high empirical support. Romanowska et al. (2005) showed that oligomycin (0.1 µM) reduced respiration by about 30% in *Pisum sativum*, and reduced mitochondrial ATP/ADP by about 78% in *Hordeum vulgare*, and decreased mitochondrial ATP synthesis by about 35% in isolated mitochondria from *P. sativum*. Vera-Vives et al. (2024) further showed in *Physcomitrium patens* ndufa5 knockout mutants deficient in mitochondrial Complex I had a 2.7-fold slower rise in cytosolic ATP during illumination than in the wild-type.

The empirical support for KER#3772 (Decrease in ATP production leads to Decrease in individual growth) is considered low, reflecting a lack of studies simultaneously measuring both ATP levels and individual growth in multicellular primary producers under a single stressor exposure. In Lemna minor exposed to 3,5-dichlorophenol, inhibition of oxidative phosphorylation (EC50 = 1.41 mg/L) co-occurred with reductions in frond area (EC50 = 1.45 mg/L) and frond number (EC50 = 2.20 mg/L), providing indirect dose-response concordance between cellular energy status and individual growth (Xie et al., 2018). Direct paired measurements of ATP and individual growth in a single primary producer remain a study gap and represent a priority for future empirical development of this KER. Nevertheless, indirect quantitative evidence from metabolic-inhibitor studies can help define plausible scaling relationships. For example, in Lemna minor exposed to 3,5-dichlorophenol, the EC50 for oxidative phosphorylation inhibition (1.41 mg/L) was close to the EC_50_ for frond area reduction (1.45 mg/L) and lower than the EC_50_ for frond number reduction (2.20 mg/L), suggesting quantitative concordance between impaired energy metabolism and growth-related endpoints (Xie et al., 2018).

The empirical support for KER#2169 (Decrease in individual growth leads to Decrease in population growth rate) is considered high, based on hierarchical ecological evidence and empirical co-occurrence of individual and population growth endpoints in standard ecotoxicological test systems. In *Lemna minor*, Xie et al. (2018) reported that 7-day exposure to 3,5-dichlorophenol caused concentration-dependent reductions in individual growth endpoints including frond area (EC_50_ = 1.45 ± 0.13 mg/L) and dry mass (EC_50_ = 1.92 ± 0.27 mg/L), which is lower than with frond number (EC_50_ = 2.20 ± 0.01 mg/L), the standard population growth rate metric in the OECD TG221 (OECD, 2006), suggesting that there is significant developmental consistency between individual growth and population growth in multicellular macrophytes.

#### 4.3.1 Overall WoE considerations

The AOP is biologically plausible and mechanistically conserved across primary producers. Empirical support is strong for the upstream KERs (MIE to photosynthesis) based on well-documented dose-response and temporal concordance, and moderate to high for most downstream KERs. The KER linking decreased ATP production to decreased individual growth currently has low empirical support, reflecting a lack of studies simultaneously measuring both endpoints in multicellular primary producers, and represents a priority for future targeted studies.

### 4.4 Uncertainties, inconsistencies, and critical gaps

AOP#567 is biologically coherent and based on mechanistic understanding of PSII inhibition and cellular bioenergetics, but important uncertainties remain. The main upstream uncertainty concerns KER#3557 (Decrease in photosynthesis leads to Decrease in mitochondrial oxidative phosphorylation), which is biologically plausible but context dependent. Decreased photosynthesis can constrain mitochondrial metabolism through reduced substrate supply and altered redox balance, but mitochondrial respiration may also be maintained or reallocated as part of acclimation under some stress conditions. Therefore, KER#3557 is retained as a biologically supported intermediate relationship, but its confidence is considered moderate and its quantitative behaviour is expected to vary with organism, exposure duration, physiological state, and compensatory capacity. The most significant empirical gap is the KER linking decreased ATP production to decreased individual growth (KER#3772). No study has simultaneously measured both ATP levels and individual growth in multicellular primary producers under a single stressor exposure, and this represents a priority for future targeted experimental work. A conceptual uncertainty exists regarding the empirical distinction between individual growth (AO#1521) and population growth rate (AO#360) in unicellular primary producers such as microalgae. Although the two AOs are retained as separate events in AOP#567, the measured endpoints may overlap in unicellular systems because cell division is closely linked to population increase. This distinction is clearer in multicellular primary producers such as macrophytes and seagrasses, where biomass accumulation, tissue expansion, frond or shoot development, and population expansion can be measured more separately. Major critical gaps remain, including the lack of integrated multi-KE dose-response datasets generated within single studies, limited species-specific parameterisation for population-level modelling, and insufficient resolution of the contribution of ROS-mediated secondary damage to downstream energy dysfunction and growth impairment. Addressing these gaps would substantially strengthen the quantitative understanding, predictive performance, and regulatory applicability of AOP#567.

### 4.5 Quantitative understanding

The quantitative understanding of AOP#567 is patchy across the pathway with strong characterisation of the photochemical and bioenergetic linkages and lack of full integration from the molecular initiating event (MIE) to the adverse outcome (AO). The relationship between QB-site binding and reduced PSII efficiency (KER#3556) is supported by concentration–response studies of PSII inhibitors, including the nanomolar potency of diuron and the characteristic sigmoidal decrease in F_v_/F_m_ with increasing exposure. However, no quantitative relationship has been established that links the fraction of occupied QB sites to the magnitude of PSII efficiency loss (Battaglino et al., 2021; Broser et al., 2011). The relationship between decreased PSII efficiency and reduced photosynthesis (KER#2333) has stronger quantitative support. Endpoints that are based on fluorescence and oxygen-evolution or carbon-assimilation endpoints tend to exhibit equivalent sensitivities and numerous studies indicate that PSII efficiency is a primary upstream parameter of impaired photosynthetic performance (U.S. EPA, 2003; Genty et al., 1989; Thomas et al., 2020). Quantitative support for the mid-pathway link between reduced photosynthesis and decreased mitochondrial OXPHOS (KER#3557) is more limited. Chemical evidence from *Chlamydomonas reinhardtii* demonstrates that inhibition of photosynthesis by Diuron or glycolaldehyde decreases light-enhanced dark respiration, supporting the hypothesis that disruption of photosynthetic processes can lead to downstream impairment of cellular respiration (Xue et al., 1996). However, there is a lack of paired dose-response data directly correlating PSII-inhibitor-induced photosynthetic decay to mitochondrial OXPHOS degradation. The OXPHOS-to-ATP relationship (KER#3558) is mechanistically well established through chemiosmotic coupling and ATP synthase activity (Mitchell, 1961; Nicholls and Ferguson, 2002). Under moderate inhibition, changes in respiratory activity are expected to translate into reduced ATP production, although pathway-specific quantitative functions for primary producers are still limited. The ATP-to-growth relationship (KER#3772) shows some quantitative scaling, with ATP depletion occurring at lower concentrations than growth inhibition in *Chlamydomonas reinhardtii* (Nestler et al., 2012). This supports ATP reduction as an upstream bioenergetic predictor of impaired growth, but the available data remain insufficient to define a general predictive function across taxa and exposure conditions. Overall, quantitative evidence for KERs are only partially supportive for full predictive qAOP modelling, thus illustrating that multi-KE dose-response data sets are needed.

For KER#3772, indirect quantitative evidence may be derived from studies that measure cellular energy impairment and growth endpoints under the same exposure. Although these studies do not provide a direct ATP-to-growth function, they inform candidate qAOP relationships by comparing effect concentrations for energy-metabolism endpoints with those for frond area, biomass, cell division, or other individual-growth metrics. Such evidence should be treated as indirect support until paired ATP and growth measurements are available in primary producers exposed to PSII inhibitors.

### 4.6 Environmental relevance

AOP#567 is of environmentally relevant concern as it corresponds to the known mode of action of PSII inhibitors (e.g. triazines, atrazine, phenylureas, diuron, triazinones and relatives) which are widely used in the agricultural and antifouling fields. These substances are widely used in agricultural settings, and some PSII inhibitors also have non-agricultural uses, including antifouling or other biocidal applications. They are frequently detected in surface waters receiving agricultural runoff, stormwater, or wastewater inputs, sometimes at concentrations close to or within effect ranges for aquatic primary producers (U.S. EPA, 2022; Wilkinson et al., 2015). Many PSII-inhibiting herbicides have also been identified as important risk drivers when measured environmental exposures are compared with ecological safety thresholds for aquatic organisms, including primary producers (Spilsbury et al., 2020). The QB binding site in the D1 protein is highly conserved in all oxygenic phototrophs including freshwater and marine microalgae, macrophytes and higher plants, suggesting a broad taxonomic applicability (Alfonso et al., 1996; Broser et al., 2011). Primary producers form the basis of ecosystem function, and long-term inhibition of PSII can lead to decreased carbon and biomass accumulation as well as population growth effects that can potentially transplant to food-web structures (Forbes and Calow, 2002). Field and mesocosm experiments have demonstrated the effects of PSII herbicides to alter the composition of aquatic community and inhibit photosynthetic function at environmentally relevant concentrations (Wilkinson et al., 2015). Because this inhibition of PSII is quick and quantifiable by means of chlorophyll fluorescence techniques, early key events are sensitive indicators of sublethal environmental stress (Maxwell and Johnson, 2000). Accordingly, AOP#567 provides a mechanistically anchored framework for assessing ecological risks of PSII inhibitors in aquatic and terrestrial environments.

## 5 Domains of applicability

### 5.1 Taxonomic applicability

**Figure 3:**
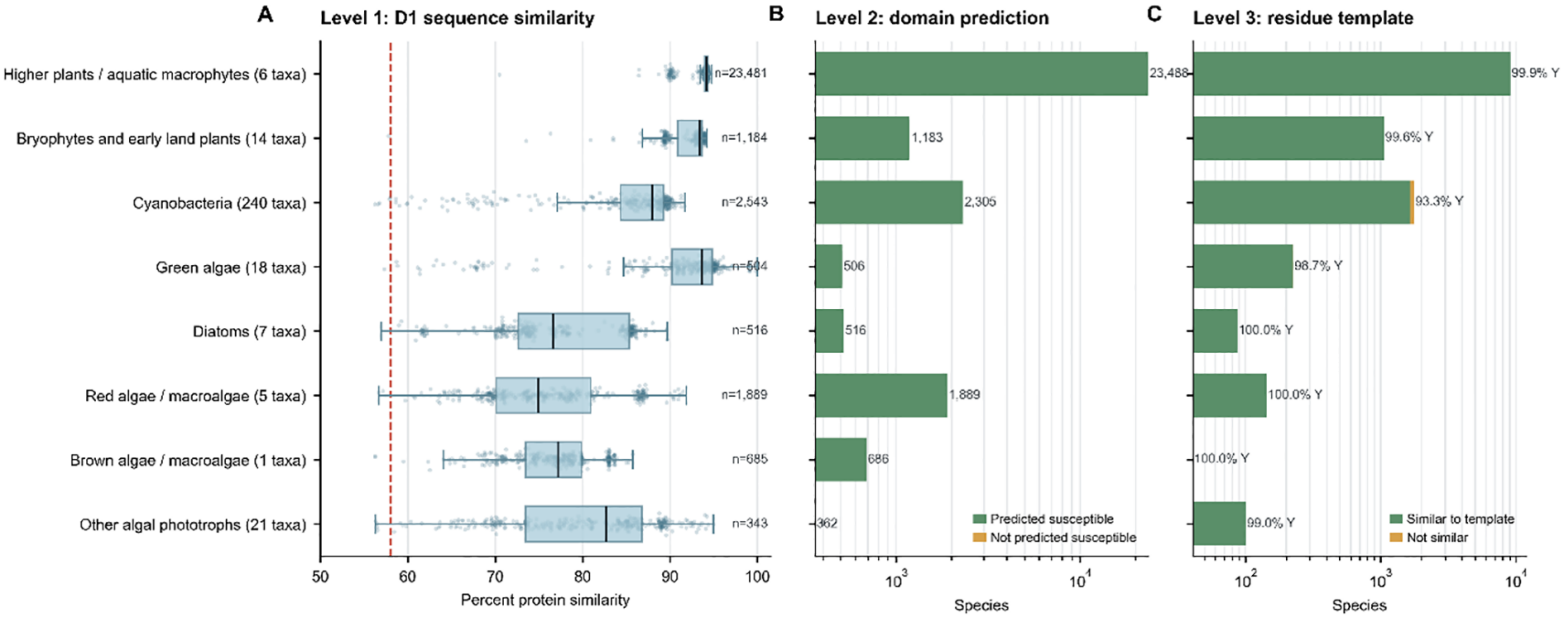
SeqAPASS evidence for conservation of the PSII D1 target across taxonomic groups. Y-axis represented major phylogenetic and functional groups of photosynthetic organisms, with the total number of analyzed taxa per group. **(A)** SeqAPASS Level 1 analysis showing percent similarity distributions for individual D1 protein sequences grouped by PSII-containing functional/ecological groups. Boxes show the interquartile range and median; points show sampled individual sequences. The red dashed line indicates the default Level 1 similarity cut-off of 56.27%. **(B)** SeqAPASS Level 2 taxon-level similarity summary for the same groups. Bars show mean percent similarity and points show median percent similarity; labels indicate the number of species. **(C)** SeqAPASS Level 3 analysis of key amino-acid positions associated with the susceptibility template. Bars show the number of species classified as similar or not similar to the template, plotted on a log scale; labels indicate the percentage classified as similar.

AOP#567 is expected to be broadly applicable across oxygenic photosynthetic organisms at the level of molecular susceptibility, because the molecular initiating event (MIE), binding of a PSII inhibitor to the QB site of the D1 protein, targets a conserved component of Photosystem II (Broser et al., 2011; Battaglino et al., 2021). These domains cover freshwater and marine microalgae, cyanobacteria, diatoms, macroalgae, aquatic macrophytes, sea grasses and higher plants. Organisms that do not have PSII are not considered to be directly impacted by this MIE, but they could be indirectly impacted by primary production changes and food-web structure (Forbes and Calow, 2002). SeqAPASS, a tool that evaluates the conservation of molecular targets across taxa using protein sequence, functional domain, and key amino acid residue analyses, supported broad conservation of the D1 protein among PSII-containing organisms (**Figure 3**). Using Chlamydomonas reinhardtii photosystem II protein D1 (NP_958377.1) as the query sequence in SeqAPASS v9.1. SeqAPASS Level 1 analysis showed broad conservation of the D1 protein across the main PSII-containing ecological groups, with most sequences above the Level 1 similarity cut-off. Conservation was supported across primary producers, including higher plants/aquatic macrophytes, cyanobacteria, red algae/macroalgae, bryophytes and early land plants, brown algae/macroalgae, diatoms, green algae and other algal phototrophs. Together, these groups comprised 31,145 Level 1 sequence records across 312 assigned taxa, supporting conservation of the D1 target across both eukaryotic primary producers and prokaryotic oxygenic phototrophs. SeqAPASS Level 2 (functional domain) and Level 3 (key amino acid residues) analysis further supported taxon level conservation, where 110 level 2 taxonomic groups were identified to display sufficient similarity to the query sequence, whereas level 3 demonstrated sufficient amino acid similarity in 300 of 326 taxonomic groups available in the supporting database (i.e. NCBI Conserved Domain Database, CDD), representing 12543 of 12683 species (98.9%). The presence of cyanobacteria in the search results of SeqAPASS gives further evidence to the hypothesis that the prokaryotic oxygenic phototrophs are also represented in the taxonomic field of applicability. This is consistent with the strong functional conservation of PSII and the D1/QB target site across cyanobacteria, where the QB-binding niche serves the same essential role in plastoquinone-mediated electron transport. As a result, inhibition of electron transfer through QB site binding is expected to occur via a similar mechanism across taxa (Allen et al., 1983; Broser et al., 2011). Variations in sensitivity with taxa are thus likely to represent biological and physiological modifiers as opposed to a lack of the molecular target (i.e. the MIE). Other modifiers that may be relevant include pigment composition, light-harvesting strategy, PSII repair capacity, tissue structure, uptake and detoxification capacity, metabolic flexibility and growth rate. For example, vascular plants and seagrasses rely on PSII for photosynthetic electron transport and exhibit pronounced decreases in PSII yield following exposure to PSII-inhibiting herbicides such as atrazine and diuron, demonstrating conservation of the herbicide target and response across taxa (Wilkinson et al., 2015). The plausible taxonomic domain of applicability encompasses green algae, diatoms, cryptophytes, euglenophytes, dinoflagellates, macroalgae, and Symbiodiniaceae, all of which possess functional PSII and are therefore expected to exhibit the molecular initiating event described in this AOP (Allen et al., 1983; Thomas et al., 2020; Flores et al., 2021).

However, conservation of the D1 protein and QB-binding residues should not be interpreted as evidence of equivalent organism-level sensitivity across all taxa. Sequence conservation supports the potential for molecular interaction with PSII inhibitors, but whole-organism responses may differ because of a number of factor including, but not limited to species-specific differences in pollutant uptake, exposure route, metabolism or detoxification, PSII repair capacity, growth rate, life stage, and physiological compensation. Therefore, the taxonomic applicability domain of AOP#567 is strongest for identifying taxa with potential molecular susceptibility to PSII inhibitors, while quantitative extrapolation of sensitivity among species requires additional consideration of exposure conditions and organism-specific physiology.

### 5.2 Sex and life stage

The applicability of AOP#567 is considered independent of sex, as the molecular initiating event targets a conserved component of the photosynthetic apparatus that is not known to exhibit sex-specific differences. The MIE and early downstream KE are founded upon photosynthesis physiology, not sex-specific biology. In photosynthetic taxa that reproduce sexually, this AOP is relevant to both males and females, gametophytes or reproductive organs where the cells or tissues have functional PSII. It is also applicable to asexually reproducing taxa, such as most algae and aquatic macrophytes. Nevertheless, the reproductive mode and reproductive state can have subsequent effects on the organism and population level by impacting vegetative growth, gamete production, spore development, seedling development, or propagation of a clone.

Life-stage applicability is associated with the presence and functional significance of PSII. Photosynthetically active life stages such as vegetative algal cells, propagules or photosynthetic spores of cyanobacteria, plant fronds, leaves, shoots, established seedlings, fall within the realm of applicability. Conversely, stages which are not yet dependent upon photosynthesis, e.g. embryos, non-growing seeds till emergence, below-ground germinating seeds before emergence, non-photosynthetic resting stages, should be less directly sensitive to triggering the MIE until PSII-containing tissues can become active. The initial stages of photosynthesis may be particularly vulnerable to disruption during periods of rapid growth, when energy requirements are high and physiological compensation is limited by short-term energy reserves or reduced adaptive capacity. Therefore, AOP#567 is expected to apply regardless of sex or reproductive strategy in PSII-containing organisms. However, the magnitude of response may differ among life stages, reflecting variation in photosynthetic demand, energy requirements, and developmental dependence on PSII-mediated electron transport.

## 6 Biological context

The AOP is applicable under both controlled laboratory conditions and environmentally relevant exposure scenarios, as the underlying biological mechanisms are expected to operate across these contexts. Although increased concentration (or doses) are typically applied in controlled experiments to determine mechanistic relationships, environmental-relevant, sublethal concentrations have been reported to affect PSII efficiency, photosynthesis and growth in aquatic systems (Wilkinson et al., 2015; Hanson et al., 2023). The persistence and mobility of PSII inhibitors in surface waters and groundwater increase the likelihood of sustained exposure in aquatic ecosystems, making chronic exposure scenarios particularly relevant for the application of this AOP.

## 7 Conclusion

As the first formally developed AOP focused on primary producers in AOPwiki. the AOP#567 described PSII inhibitors inhibit the growth of primary producers by binding to D1 proteins at the Q_B_ site and thus reduce photosynthesis and mitochondrial bioenergetics. Weight-of-evidence assessment based on modified Bradford-Hill considerations demonstrates high biological plausibility and empirical support for upstream photochemical KERs, while downstream bioenergetic KERs exhibit greater context dependency, with a critical gap in paired ATP-growth endpoint data for primary producers. SeqAPASS analysis verified broad conservation of the D1 protein and QB-binding residues across major photosynthetic lineages, supporting a wide taxonomic applicability domain. The AOP#567 provides a transparent, evidence-based mechanistic framework to support hazard identification, cross species extrapolation, and ecological risk assessment of PSII inhibiting herbicides across aquatic and terrestrial ecosystems.
